# The Small Auxin Upregulated RNA *PsnSAUR6* from *Populus simonii × P. nigra* Enhances Drought Tolerance in Transgenic Tobacco

**DOI:** 10.3390/plants15091398

**Published:** 2026-05-02

**Authors:** Shuang Liu, Xin Sun, Lei Wang, Fengqingyang Chen

**Affiliations:** Department of Biotechnology, Institute of Advanced Technology, Heilongjiang Academy of Sciences, Harbin 150001, China

**Keywords:** *Populus simonii × P. nigra*, drought stress, small auxin up RNA (*SAUR*) gene, ABA signaling

## Abstract

Intensifying drought stress under global climate change poses a significant threat to woody plants, highlighting the critical need to identify key genes conferring drought tolerance. Here, we characterized *PsnSAUR6*, a Small Auxin Upregulated RNA (*SAUR*) family gene from poplar (*Populus simonii × P. nigra*) that is responsive to drought and abscisic acid (ABA). Overexpression of *PsnSAUR6* in transgenic tobacco conferred superior drought tolerance, evidenced by increased biomass, enhanced root elongation, improved stomatal regulation, and favorable physiological responses, including higher proline content and peroxidase (POD) activity but lower malondialdehyde (MDA). Transcriptome analysis revealed that under water deficit, *PsnSAUR6* suppressed the ABA negative regulator *PP2C37* while upregulating key antioxidant defense-related transcription factors (*ERF020*, *NAC83*, *MYB2*) and the potassium transporter *HAK5*. Collectively, these findings establish *PsnSAUR6* as a positive regulator in ABA-mediated drought adaptation, presenting it as a promising genetic target for enhancing the climate resilience of woody plants.

## 1. Introduction

Natural ecosystems and human habitats are seriously threatened by the recent sharp rise in the frequency and severity of droughts brought on by global warming. CO_2_ is the primary contributor to global warming, accounting for the highest proportion and pace among human-caused greenhouse gases. Carbon dioxide is typically absorbed by plants through photosynthesis, particularly by woody plants. Approximately two-thirds of the carbon fixed annually by the terrestrial ecosystem is estimated to be sequestered by global forest systems. Therefore, the resilience of woody plants under drought stress directly influences the global carbon cycle and climate stability.

Prolonged drought triggers a range of physiological and morphological changes in plants, including growth inhibition, leaf wilting, stomatal closure, and reduced photosynthetic capacity [1,2]. Severe drought can lead to plant mortality, drastically diminishing the carbon sequestration potential of forest ecosystems. Understanding the molecular mechanisms underlying drought resistance in woody plants is thus essential for enhancing forest resilience and mitigating climate impacts.

Under drought stress, plants undergo morphological and structural adjustments regulated by multiple hormones. Auxin, a key regulator of plant growth and development, plays a vital role in drought-induced morphological remodeling. It rapidly modulates downstream gene expression and synergizes with abscisic acid (ABA) to regulate stomatal aperture and minimize water loss [3,4]. Under sustained stress, auxin polar transport directs cell division and development, playing a role in plant morphogenesis. For example, in the aboveground sections, it reduces water loss by limiting stem elongation and decreasing leaf surface area [5,6], whereas in the subsurface parts, it regulates root architecture dynamically by changing auxin distribution and transport. Specifically, under mild drought, the auxin signaling pathway stimulates primary root extension as well as the multiplication of lateral roots and root hairs, directing roots toward deeper and denser development in order to efficiently expand their water-absorbing range [7,8,9]. However, as drought intensity increases, auxin and ABA signaling pathways work together to direct the root system’s growth focus away from lateral expansion and toward vertical elongation [10,11,12].

Early auxin response genes comprise three major gene families: *Aux/IAA* (*Auxin/indole-3-acetic acid*)*, GH3* (*Gretchen hagen 3*), and *SAUR* (*small auxin upregulated RNA*) [13]. The *SAUR* gene family has the most members and was discovered in the hypocotyl of soybeans (*Glycine max*) in 1987 [14]. These *SAUR* genes are found in a wide range of plants, including *Arabidopsis thaliana* [15], rice [16], and sorghum [17], as well as economic forest trees such as *Populus tomentosa* [18], apple [19], and loquat [20]. The *SAUR* genes are often found in clusters within genomes, with the majority missing introns. The encoded mRNA molecules are extremely unstable, capable of fast production and degradation in response to auxin stimulation. This property enables *SAUR* genes to respond quickly to auxin signals, regulating a variety of activities in plant growth and development. At the protein level, SAUR proteins directly modulate cell wall acidification by inhibiting PP2C.D-class phosphatase activity, thereby alleviating its inhibitory phosphorylation of the proton pump H^+^-ATPase and promoting cell elongation [15]. For example, in cucumber (*Cucumis sativus* L.), overexpression of *CsSAUR31* enhances both root and hypocotyl elongation [21]. Similarly, in wheat (*Triticum aestivum* L.), overexpression of *TaSAUR66-5B* not only stimulates root growth but also increases biomass and grain yield in transgenic plants [22]. Beyond growth regulation, *SAUR* genes are involved in diverse developmental processes such as light-mediated cotyledon and apical hook opening [23,24], floral organ formation [25,26], and fruit development [27,28].

In addition to their roles in growth and development, *SAUR* genes have attracted increasing attention in recent years for their involvement in drought responses. Under drought conditions, the expression levels of *SAUR* genes undergo dynamic changes. By integrating auxin signaling, they modulate plant growth patterns to enhance stress adaptation. For instance, in *Zygophyllum xanthoxylum*, overexpression of *ZxSAUR15* promotes root development in *Arabidopsis* and improves drought tolerance [29]. Conversely, in peanut, transgenic *Arabidopsis* plants overexpressing *AhSAUR3* show reduced survival under drought stress compared to wild-type plants [30]. Similarly, in *Arabidopsis*, overexpression of *PtSAUR8* from hybrid poplar (*Populus × euramericana* cv. “Nanlin95”) enhances stomatal closure upon ABA treatment, thereby increasing drought tolerance [31]. Furthermore, He et al. demonstrated that *Arabidopsis AtSAUR32* regulates ABA sensitivity by interacting with PP2C Class A proteins AtHAI1 and AtAIP1, consequently enhancing drought resistance [32]. These findings collectively suggest that *SAUR* genes may act as a molecular link connecting auxin-mediated growth regulation with drought adaptive responses. However, research in this field has predominantly centered on herbaceous species, whereas investigations in woody plants remain limited and largely confined to model species such as *Populus trichocarpa* [18] and *Vitis vinifera* [33]. Most of these studies are still at a foundational stage, focusing on genome-wide identification and preliminary expression profiling. A systematic understanding of *SAUR* gene response patterns to drought, their regulatory targets, and their interaction with other hormonal pathways in woody plants remains notably incomplete.

*Populus simonii × P. nigra* (a hybrid poplar) was artificially bred by the Forestry Science Research Institute of the Chinese Academy of Forestry. This hybrid combines the stress tolerance of *P.simonii* with the rapid growth of *P. nigra*, maintaining stable growth advantages under dry and cold conditions. These characteristics make it an excellent model system for investigating the physiological and molecular mechanisms underlying drought stress responses in woody plants [34,35]. However, research on the *SAUR* gene family in poplars remains in its early stages, primarily limited to genome-wide identification and stress-related expression profiling of selected members [18]. The specific functions of *SAUR* genes in poplar stress adaptation and their underlying molecular regulatory mechanisms are still largely unknown. In our previous work, an unannotated transcript responsive to drought was identified via RNA-seq analysis across the roots of *Populus simonii × P. nigra* under drought stress, and this transcript was found to be significantly downregulated (log_2_FoldChange = −4.56). This transcript showed high sequence similarity to *Populus tomentosa SAUR6* (*Potri.002G000600*). Following the nomenclature established by Hu et al. [18], we designated this gene *PsnSAUR6*. This study is the first to validate the involvement of *SAUR* genes in the drought-stress response of *Populus simonii × P. nigra*, their sensitivity to ABA, and the underlying molecular mechanisms. Elucidating the function and regulatory mechanism of *PsnSAUR6* under drought stress not only provides a genetic resource for enhancing poplar drought tolerance but also offers valuable insights for molecular breeding of stress-resistant forest trees.

## 2. Results

### 2.1. Cloning and Sequence Analysis of PsnSAUR6

**Figure 1:**
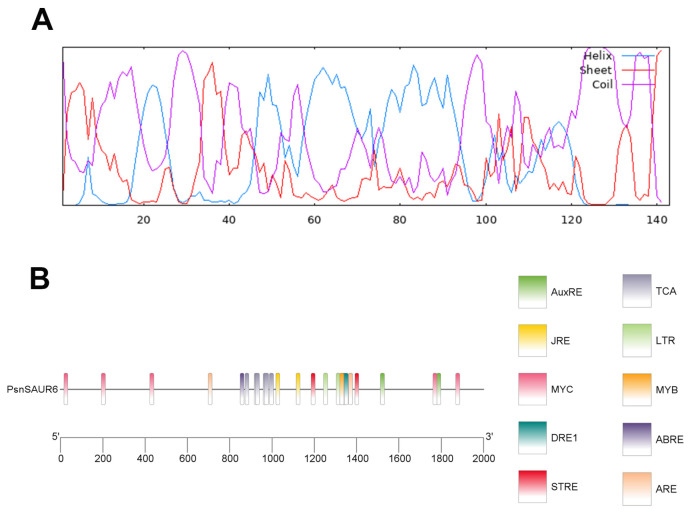
Bioinformatic characterization of *PsnSAUR6*. (**A**): Secondary structure prediction. The structure is dominated by α-helices (35.66%) and random coils (51.05%), with extended strands comprising 13.29%. The prediction was performed using GOR4. (**B**): *Cis*-regulatory element analysis of the promoter region. The promoter contains elements associated with growth regulation, abscisic acid signaling, dehydration, and low-temperature responses. Prediction was performed using PlantCARE.

**Figure 2:**
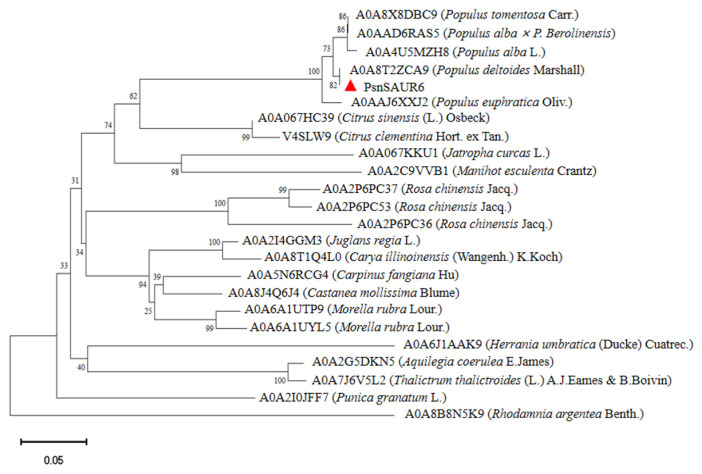
Phylogenetic analysis of PsnSAUR6 and related SAUR proteins. The red triangle highlights the position of PsnSAUR6. The evolutionary tree was constructed using the Neighbor-Joining method in MEGA11, based on homologous sequences filtered by an E-value cutoff of <1 × 10^−10^. Genetic distances were computed using the p-distance model. Node stability was assessed with 1000 bootstrap replicates; values ≥ 50% are shown. The scale bar indicates amino acid substitutions per site.

The full-length cDNA of *PsnSAUR6* was amplified by RT-PCR using the cDNA of leaves of *Populus simonii × P. nigra* as template, and a band of about 500 bp was obtained. Sequencing and ORF analysis confirmed that the *PsnSAUR6* gene lacks introns, comprising a 137 bp 5′ UTR, a 432 bp coding sequence (CDS), and a 168 bp 3′ UTR. The CDS encodes a polypeptide of 143 amino acids, with leucine (Leu) being the most abundant residue (12.5%). The protein contains 15 negatively charged (Asp + Glu) and 10 positively charged (Arg + Lys) residues. The deduced amino acid sequence of *PsnSAUR6* is provided in the Supplementary Materials. The molecular weight of the protein encoded by *PsnSAUR6* was 16.060 kDa, and the theoretical isoelectric point (pI) was 6.08 (Table S2). The secondary structure of PsnSAUR6 was dominated by α-helices (35.66%) and random coils (51.05%), while extended strands comprised only 13.29% (Figure 1A). The promoter of *PsnSAUR6* was predicted to contain *cis*-elements related to growth as well as various abiotic stress responses, such as dehydration, abscisic acid signaling and low-temperature adaptation (Figure 1B). The phylogenetic tree indicated its closest relationship is with *Populus deltoides* (98.68% identity), followed by other poplar species, while it is more distantly related to *Jatropha curcas*, *Citrus sinensis*, and *Juglans regia* (Figure 2).

### 2.2. Response of PsnSAUR6 to PEG6000-Induced Osmotic Stress

Our previous transcriptome analysis revealed that *PsnSAUR6* was responsive to osmotic stress. Furthermore, an analysis of its promoter region identified several drought-related *cis*-acting elements. These findings led us to hypothesize that *PsnSAUR6* functions in the drought stress response pathway. Consequently, we examined its expression patterns in various tissues (roots, stems, and leaves) of poplar cuttings under simulated osmotic stress induced by PEG6000 at −0.7 MPa.

**Figure 3:**
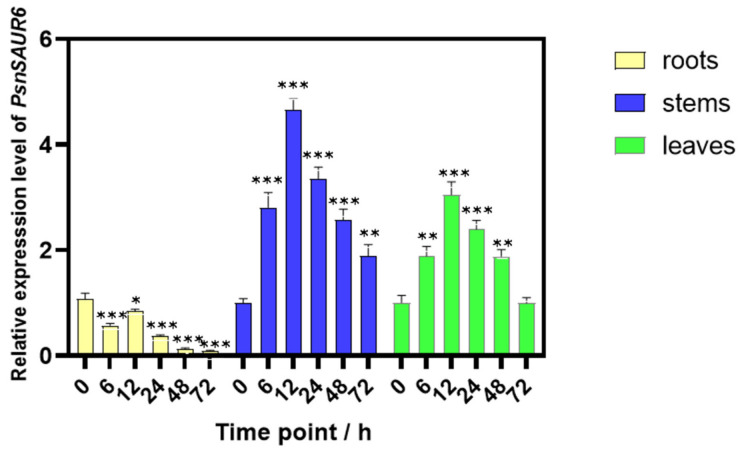
Organ-specific expression of *PsnSAUR6* in *Populus simonii × P. nigra* under water deficit. The asterisk indicates a statistically significant difference compared to non-genetically modified tobacco, Student’s *t*-test, * indicates *p* < 0.05, ** indicates *p* < 0.01, *** indicates *p* < 0.001.

The expression of *PsnSAUR6* exhibited a organ-specific pattern, with the highest transcript levels detected in the stems, followed by the leaves, and the lowest in the roots. This pattern was consistent under both control and osmotic stress conditions (Figure 3). Following osmotic stress, the expression of *PsnSAUR6* showed divergent responses across different tissues. In the roots, its expression was significantly downregulated at all time points compared to the control (0 h). In contrast, *PsnSAUR6* expression was significantly upregulated in the stems at all sampling time points. A similar trend of upregulation was observed in the leaves, except at 72 h, where the expression level was not significantly different from the control. These findings indicate that *PsnSAUR6* plays an organ-specific role in the response of poplar to osmotic stress.

### 2.3. Overexpression of PsnSAUR6 Enhances Drought Tolerance in Transgenic Tobacco

**Figure 4:**
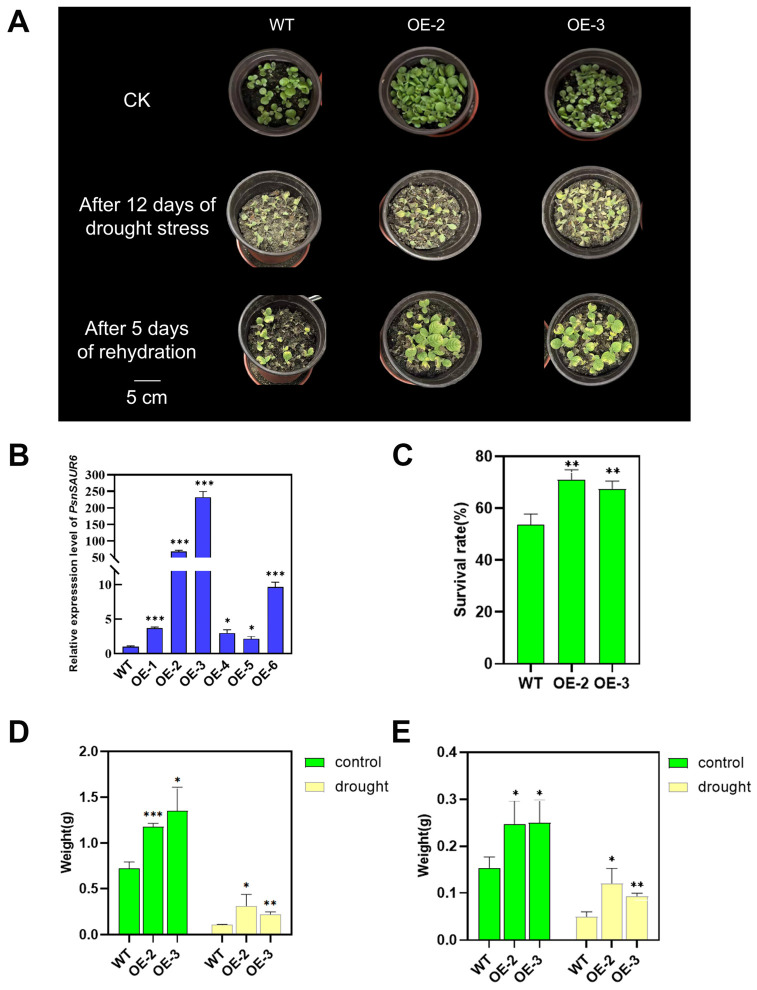
Drought response of *PsnSAUR6*-overexpressing and wild-type tobacco. (**A**): Phenotypic comparison during drought stress and re-watering. (**B**): *PsnSAUR6* transcript levels in T_2_ transgenic lines. (**C**): Survival rates post-drought. (**D**,**E**): Biomass (fresh and dry weight) of aerial tissues (WT: Non-transgenic tobacco; OE-1 to OE-6: Different transgenic lines. The asterisk indicates a statistically significant difference compared with non-transgenic tobacco, Student’s *t*-test, * indicates *p* < 0.05, ** indicates *p* < 0.01, *** indicates *p* < 0.001).

To investigate the function of *PsnSAUR6* in response to drought stress, we generated transgenic tobacco plants overexpressing this gene. Following antibiotic screening and quantitative real-time PCR (qRT-PCR) validation, six independent transgenic lines were successfully obtained (Figure 4B). Two lines, OE-2 and OE-3, which exhibited the highest levels of *PsnSAUR6* expression, were selected for subsequent drought tolerance assays.

Three-week-old transgenic and wild-type (WT) plants were subjected to drought stress by withholding water for 12 days, followed by a 5-day rehydration period. After the drought treatment, both transgenic and wild-type plants exhibited water shortage symptoms such as leaf wilting, curling, and chlorosis. However, the transgenic lines exhibited a noticeably milder phenotype than WT (Figure 4A). Upon rewatering, the transgenic plants exhibited a superior recovery capacity, characterized by greater leaf turgor. Quantitative analysis revealed that the survival rates of the transgenic lines OE-2 (71.11%) and OE-3 (67.5%) were significantly higher than that of WT (53.57%) after the drought and recovery cycle (*p* < 0.01) (Figure 4C). Furthermore, biomass measurements of 4-week-old plants indicated that under both normal and drought conditions, the aerial parts fresh weights (FW) and dry weights (DW) of the transgenic lines were significantly greater than those of WT (*p* < 0.05) (Figure 4D,E).

**Figure 5:**
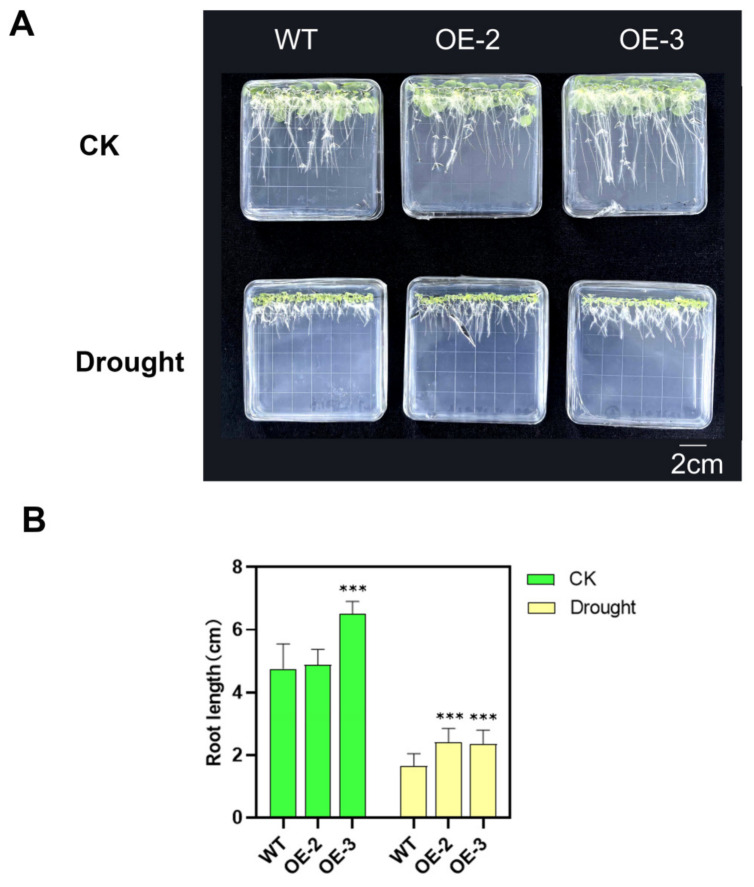
Enhanced root growth of *PsnSAUR6*-overexpressing tobacco under osmotic stress. (**A**): Phenotype of seedlings grown on MS medium with or without mannitol at −0.5 MPa for 10 days; (**B**): Column chart of vertical root length of transgenic tobacco and non-transgenic tobacco. (WT: Non-transgenic tobacco; OE-2 and OE-3: Different transgenic lines. The asterisk indicates a statistically significant difference compared with non-transgenic tobacco, Student’s *t*-test, *** indicates *p* < 0.001).

To assess the impact of *PsnSAUR6* overexpression on root development, we measured the primary root length of seedlings grown vertically on medium for 10 days. Under control conditions, the root length of the OE-2 line was comparable to that of WT, whereas the OE-3 line exhibited significantly longer roots (*p* < 0.001). Following osmotic treatment, root elongation was inhibited in all lines. Nevertheless, both transgenic lines maintained significantly longer primary roots than the WT (*p* < 0.001) (Figure 5).

**Figure 6:**
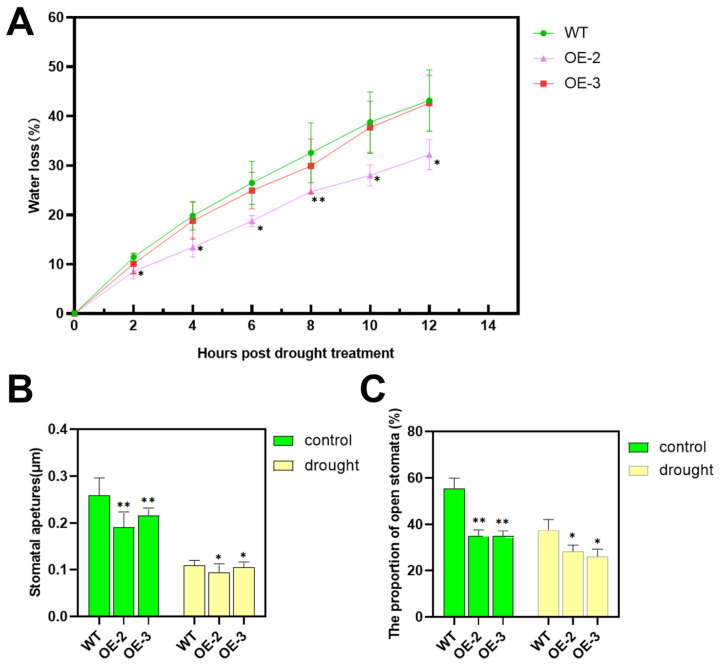
Reduced water loss and altered stomatal dynamics in *PsnSAUR6*-overexpressing tobacco under drought stress. (**A**): Percentage of water loss in isolated leaves of transgenic tobacco with *PsnSAUR6* gene under drought stress; (**B**): Stomatal aperture size (μm) in wild-type and transgenic tobacco after 3 days of drought stress; (**C**): Proportion of open stomata (%) in wild-type and transgenic tobacco after 3 days of drought stress. (WT: wild-type tobacco; OE-2 and OE-3: different transgenic lines. Data are shown as mean ± SD. The asterisk indicates a statistically significant difference compared to non-genetically modified tobacco, Student’s *t*-test, * indicates *p* < 0.05, ** indicates *p* < 0.01). Representative images of stomatal phenotypes (aperture and opening ratio) are shown in Figure S1.

To explore the role of *PsnSAUR6* in regulating water status, we tested the water loss of detached leaves. The coefficients of variation (CVs) for the initial fresh and dry weights of leaves from all lines were below 20% (Table 1), indicating that the observed differences in water loss were not attributable to initial variations in biomass. The results demonstrated that over a 12 h period, leaves from all plants progressively lost water (Figure 6A). However, the rate of water loss in transgenic plants was consistently lower than that of WT. This effect was particularly pronounced in the OE-2 line (*p* < 0.01).

To elucidate the physiological basis for the reduced water loss, we examined key stomatal characteristics in mature leaves from the same nodal position on plants used in previous water loss assays. First, we measured the proportion of open stomata in different tobacco strains within the same observation area. Under normal growth conditions, the stomatal opening rate of transgenic lines OE-2 (34.83%) and OE-3 (34.86%) was significantly lower than that of WT (55.62%) (*p* < 0.01). This pattern persisted under drought stress, with transgenic lines maintaining lower opening rates (OE-2, 28.28%; OE-3, 26.16%) compared to the WT (37.60%, *p* < 0.05) (Table S3). In addition to the opening rate, we directly measured stomatal aperture (n ≥ 50 per line). Under control conditions, the average aperture of WT (0.260 μm) was significantly wider than that of OE-2 (0.160 μm) and OE-3 (0.217 μm) (*p* < 0.01). While drought induced stomatal closure in all lines, the apertures of transgenic lines remained significantly smaller than in WT (*p* < 0.05) (Figure 6B).

**Figure 7:**
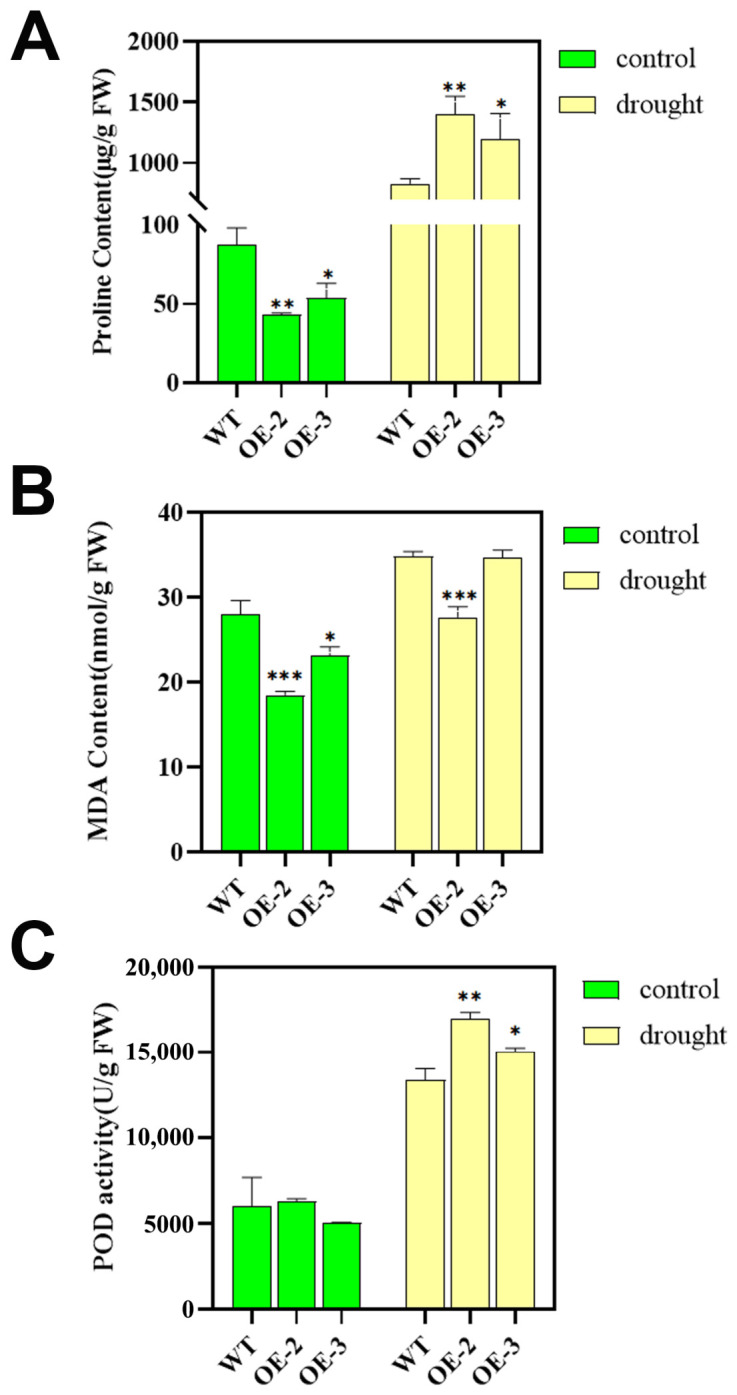
Proline, malondialdehyde (MDA), and peroxidase (POD) activity in wild-type and *PsnSAUR6* transgenic tobacco. (**A**): Proline content; (**B**): Malondialdehyde (MDA) content (estimated from thiobarbituric acid reactive substances, TBARS); (**C**): Peroxidase (POD) activity (WT: Non-transgenic tobacco; OE-2 and OE-3: Different transgenic lines. The asterisk indicates a statistically significant difference compared to non-transgenic tobacco, Student’s *t*-test, * indicates *p* < 0.05, ** indicates *p* < 0.01, *** indicates *p* < 0.001).

We next investigated the physiological and biochemical responses of the tobacco genotypes to drought. Under well-watered control conditions, the transgenic lines exhibited significantly lower proline content and malondialdehyde (MDA) content but showed no differences in peroxidase (POD) activity compared to WT. The advantages of *PsnSAUR6* overexpression became evident under drought stress. The transgenic plants accumulated significantly more proline, a key osmoprotectant, than WT *(p* < 0.05; Figure 7A). Concurrently, they displayed lower levels of MDA, suggesting reduced oxidative damage, and significantly elevated POD activity, indicating enhanced peroxidase-mediated antioxidant defense (Figure 7B, C). Notably, the OE-2 line showed the most robust response, with its proline content, MDA content and POD activity being significantly different from those of WT (*p* < 0.01), highlighting its enhanced protection against oxidative damage.

### 2.4. PsnSAUR6 Response to ABA

**Figure 8:**
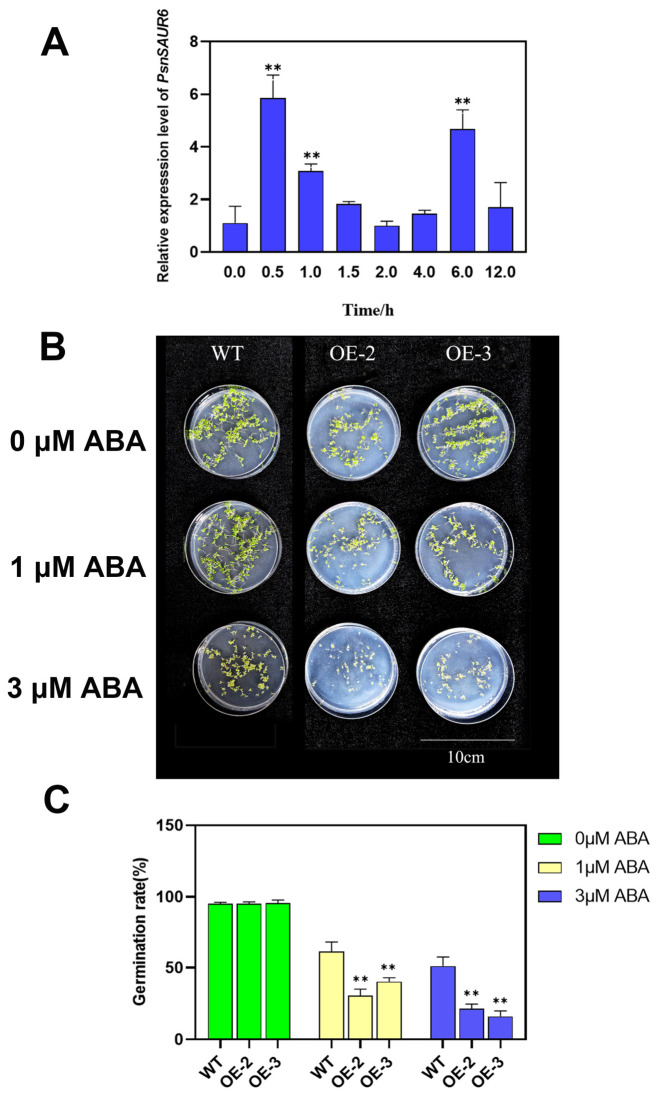
Response of *PsnSAUR6* to ABA. (**A**): Expression of *PsnSAUR6* in poplar leaves after ABA treatment; (**B**): Germination of tobacco; (**C**): Seed germination rates of non-transgenic and transgenic tobacco under ABA treatment (WT: non-transgenic tobacco; OE-2, OE-3: transgenic tobacco. The asterisk indicates statistically significant difference compared with non-transgenic tobacco, Student’s *t*-test, ** indicates *p* < 0.01).

Based on our physiological and bioinformatic analyses, we hypothesized that *PsnSAUR6* functions within the ABA signaling pathway to promote stomatal closure. To test this, we examined the expression levels of *PsnSAUR6* in poplar leaves following treatment with 100 µM ABA. Quantitative real-time PCR analysis revealed a rapid and dynamic expression pattern of *PsnSAUR6*. Transcript levels increased sharply to an initial peak at 0.5 h post-treatment before declining to baseline levels by the 2 h mark (Figure 8A). Subsequently, a second distinct peak was observed at 6 h, after which expression gradually decreased. Compared to the pre-treatment control (0 h), the upregulation of *PsnSAUR6* was statistically significant at 0.5 h, 1 h, and 6 h (*p* < 0.01).

To elucidate the role of *PsnSAUR6* in ABA-mediated germination inhibition, we assayed the germination rates of transgenic and wild-type (WT) seeds on MS medium containing a gradient of ABA concentrations (0 µM, 1 µM, and 3 µM). While all lines displayed high germination rates (>95%) under control conditions (Figure 8B,C), transgenic lines exhibited hypersensitivity to ABA. Although ABA treatment dose-dependently inhibited germination across all lines, the transgenic plants displayed significantly greater inhibition of germination (*p* < 0.01). Specifically, at 1 µM ABA, the germination rate of WT remained above 60%, whereas the OE-2 and OE-3 lines were strongly inhibited, reaching only 30.57% and 40.34%, respectively. This disparity widened further at 3 µM ABA: WT germination persisted at 51.16%, while OE-2 and OE-3 rates plummeted to 21.57% and 16.02%. These findings suggest that *PsnSAUR6* overexpression confers heightened sensitivity to ABA during seed germination.

### 2.5. Transcriptome Analysis of Transgenic Plants and Prediction of Genes Potentially Regulated by PsnSAUR6

**Figure 9:**
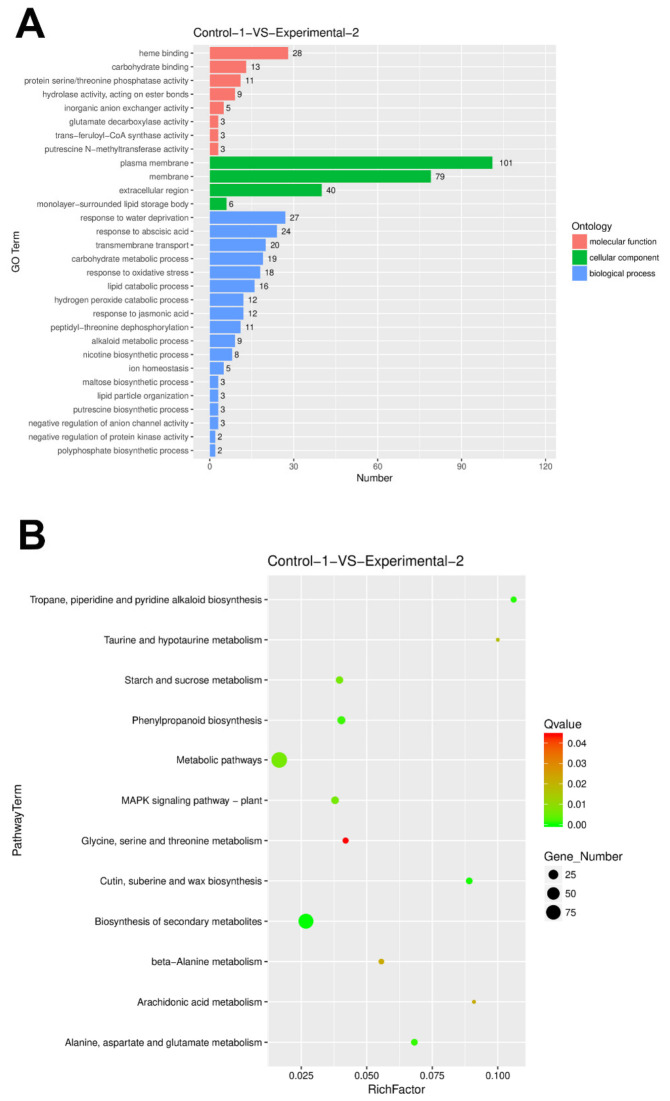
RNA-seq analysis of transgenic tobacco plants. (**A**): GO analysis of DEGs; (**B**): KEGG pathway analysis of DEGs.

To explore the molecular mechanisms underlying *PsnSAUR6* function, we performed a comparative RNA-seq analysis between transgenic and non-transgenic plants. As shown in Figure 9, the overexpression of *PsnSAUR6* resulted in 823 differentially expressed genes (DEGs), including 397 upregulated and 426 downregulated genes. Gene Ontology (GO) annotation assigned 498 DEGs to terms under the Molecular Function (MF), Cellular Component (CC), and Biological Process (BP) categories. In the MF category, DEGs were primarily enriched in heme binding, carbohydrate binding, and protein serine/threonine phosphatase activity. For the CC category, the most abundant terms were plasma membrane, membrane, extracellular region, and monolayer-surrounded lipid storage body. Regarding BP, the DEGs were mainly involved in responses to water deprivation and abscisic acid (ABA), transmembrane transport, carbohydrate metabolic processes, oxidative stress response, and lipid catabolic processes. KEGG pathway analysis mapped 256 DEGs to 12 pathways, with significant enrichment observed in metabolic pathways and the biosynthesis of secondary metabolites.

**Figure 10:**
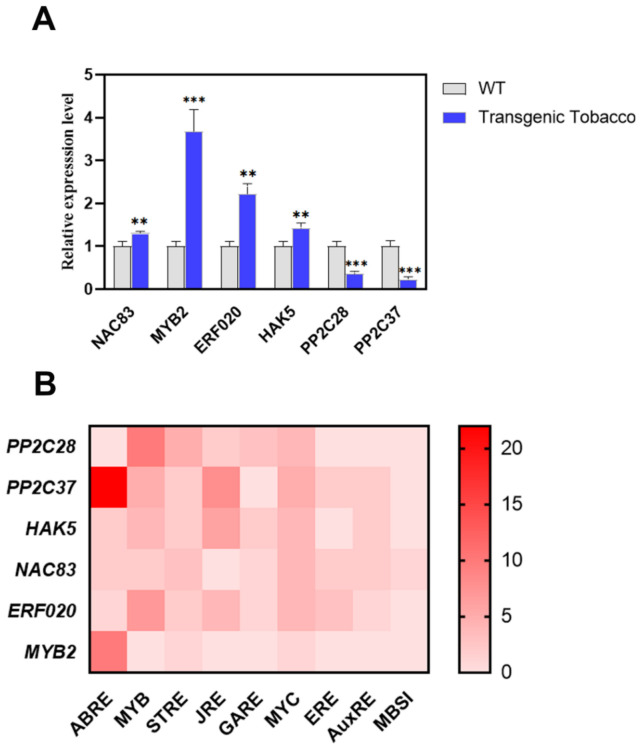
qRT-PCR validation and promoter element analysis of candidate genes identified from transcriptomics. (**A**): qRT-PCR results of RNA-seq candidate gene (asterisks indicate statistically significant differences compared to non-transgenic tobacco, Student’s *t*-test, ** indicates *p* < 0.01, *** indicates *p* < 0.001.) (**B**): Promoter *cis*-element composition of candidate genes (Color intensity corresponds to the number of identified elements per promoter.) ABRE: ABA-responsive element; MYB: MYB binding site; STRE: stress-responsive element; JRE: jasmonate-responsive element; GARE: gibberellin-responsive element; MYC: MYC binding site; ERE: ethylene-responsive element; AuxRE: auxin-responsive element; MBSI: MYB binding site I.

Further analysis of the transcriptomic data revealed significant expression changes in key transcription factors and functional proteins (Table 2). Notably, transcription factors including *MYB2, ERF020*, and NAC domain-containing protein 83 (*NAC83*) were upregulated, with log_2_ fold changes (log_2_FC) of 3.11, 2.82, and 1.27, respectively. Similarly, the expression of Potassium transporter 5 (*HAK5*) increased (log_2_FC = 1.70). Conversely, negative regulators of ABA signaling, specifically *PP2C28* and *PP2C37*, were downregulated, showing log_2_FC values of −3.14 and −2.04, respectively. The reliability of the RNA-seq data was subsequently validated by qRT-PCR analysis (Figure 10A).

To elucidate the transcriptional regulation of the candidate genes identified from our transcriptome analysis, we analyzed their promoter regions for potential *cis*-acting regulatory elements associated with hormone and stress responses. This analysis revealed that all examined differentially expressed genes (DEGs), with the notable exception of *NtaPP2C28*, contained ABA-responsive elements (ABREs) (Figure 10B). Among these ABRE-containing genes, the elements were particularly abundant in the promoters of *NtaMYB2* and *NtaPP2C37*.

Furthermore, a phylogenetic analysis was performed by comparing the amino acid sequences of these candidate proteins with their homologs in *Arabidopsis thaliana* (Figures S2 and S3). The results showed that NtaPP2C37, NtaPP2C28, NtaMYB2, NtaERF020, NtaNAC83, and NtaHAK5 share the highest sequence similarity with the *Arabidopsis* proteins AT3G11410, AT2G34740, AT3G24310, AT1G71520, AT5G13180, and AT4G13420, respectively.

## 3. Discussion

To cope with drought stress, plants have evolved sophisticated defense mechanisms at the physiological, biochemical, and molecular levels, orchestrated by a complex phytohormonal regulatory network [36]. Among these hormones, the crosstalk between auxin and ABA plays a pivotal role in modulating critical adaptive responses, such as stomatal dynamics and root system architecture remodeling [3,4]. As a primary family of early auxin-response genes, *SAURs* (*Small Auxin Up RNAs*) are characterized by an intronless structure and rapid mRNA turnover, which enables a swift response to hormonal signals [13]. Their canonical function is to promote cell elongation by directly regulating plasma membrane H^+^-ATPase activity [15]. Although recent studies have underscored the importance of *SAUR* genes in plant stress responses, this research has predominantly focused on herbaceous species [25,31]. Consequently, the functional roles of the *SAUR* family in woody perennials remain largely unexplored, particularly in a key model hybrid like *Populus simonii × P. nigra*, which is noted for its rapid growth and high stress resilience [35].

We identified and cloned *PsnSAUR6*, a drought-responsive gene from *Populus simonii × P. nigra*, through transcriptome screening. To investigate the potential role of this gene in drought resistance, we performed a series of in silico analyses. Analysis of the promoter region revealed the presence of multiple stress-related *cis*-acting elements, including dehydration-responsive, ABA-responsive, and cold-responsive elements. Analyzing the expression pattern of *PsnSAUR6* provided key insights into its potential organ-specific functions under PEG6000-induced osmotic stress. We observed *PsnSAUR6* was significantly downregulated in roots while being upregulated in stems and leaves under osmotic stress. In these aerial tissues, its expression was transiently induced, peaking at the 12th hour before declining over time (Figure 3). The rapid upregulation of the gene in stems and leaves helps to promptly initiate stress resistance regulation and reduce water loss. This pattern is consistent with other stress-responsive genes, such as rice *OsFLP*, which is rapidly induced by drought to reduce leaf water loss via the *NAC* transcription factor pathway [37]. The subsequent decline in *PsnSAUR6* expression may reflect a physiological trade-off, where the plant curtails the stress response to prevent excessive growth inhibition and conserve resources [38]. In contrast, the expression of *PsnSAUR6* in the root was inhibited by drought. This downregulation may reflect an adaptive strategy, as under severe drought, prioritizing root proliferation can be a high-risk strategy. If deep moisture is unavailable, depleting limited carbon reserves for root proliferation could be detrimental to survival [10,11,39]. We therefore speculate that repressing *PsnSAUR6* in roots enables the plant to limit root expansion and prioritize resource conservation during severe osmotic stress.

To functionally validate the role of *PsnSAUR6* in drought tolerance, we compared the performance of transgenic and wild-type (WT) tobacco lines. Under drought stress, the *PsnSAUR6*-overexpressing lines exhibited a significantly higher survival rate than their WT counterparts (Figure 4C). Notably, these transgenic lines already displayed a superior growth phenotype even under well-watered conditions, characterized by longer primary roots, increased plant height, and greater aboveground biomass (Figure 4D,E and Figure 5). Specifically, under control conditions, the primary root length of OE-2 was comparable to that of WT, whereas OE-3 exhibited significantly longer roots. This phenotypic divergence is characteristic of line-specific effects commonly observed in transgenic studies, likely attributable to variations in T-DNA integration loci and corresponding transgene expression levels [40]. Under osmotic stress conditions, the primary root lengths of OE-2 and OE-3 lines were significantly longer than those of WT. In transgenic tobacco, the constitutive expression of *PsnSAUR6* driven by the 35S promoter effectively overcame the normal suppressive effect of osmotic stress on root elongation. In a natural soil environment, this sustained root growth capacity would enable the plants to develop a more extensive root system to access deeper water, thereby translating into the superior survival advantage observed during drought stress. For instance, overexpression of *TaNAC1* in wheat promotes root elongation, while *OsSAUR11* in rice increases the ratio of deep rooting (RDR), both of which lead to improved drought resistance [16,41]. Consistent with this paradigm, we propose that *PsnSAUR6* enhances water foraging capacity by promoting primary root growth. Beyond root architecture, the increased biomass observed in transgenic lines may provide a more robust physiological foundation for withstanding environmental stress. For instance, overexpression of *BdRFS* in *Brachypodium distachyon* was shown to increase stem dry weight and relative water content, thereby enhancing its drought resistance [42]. Similarly, the overexpression of *ScMYBAS1-3* improved both total dry weight and drought tolerance in rice (*Oryza sativa L.* ssp. *japonica* cv. Nipponbare) [43]. Collectively, we suggest that *PsnSAUR6* functions as a key regulator mediating the trade-off between growth and stress adaptation, contributing to the rapid growth and high resilience characteristic of *Populus simonii × P. nigra.*

During the initial phase of drought stress, plants rapidly accumulate osmolytes, such as proline and soluble sugars. This response, coupled with the ABA signaling pathway, lowers the cellular osmotic potential to improve water uptake and enhance drought resistance. Concurrently, drought stress triggers a significant accumulation of reactive oxygen species (ROS), leading to lipid peroxidation and the generation of cytotoxic secondary metabolites like malondialdehyde (MDA), which can damage cell membranes, proteins, and nucleic acids [44,45]. To counteract this oxidative damage, plants enhance their antioxidant defense system by upregulating enzymes such as superoxide dismutase (SOD), catalase (CAT), and peroxidase (POD) to scavenge excess ROS [45]. In this study, the transgenic tobacco line OE-2 responded to drought treatment with a significant increase in proline content, a reduction in TBARS levels (as an indicator of MDA content), and enhanced POD activity relative to the wild type. This aligns well with previous literature. For instance, the overexpression of *MdATG8i* was shown to confer drought resistance in apple (*M. domestica* ‘GL-3′) by decreasing MDA and ROS content and elevating flavonoid levels [46]. In another study, Chen et al. demonstrated that *PtSAUR8* overexpression improved drought tolerance by boosting proline accumulation while reducing cellular ROS, MDA, and hydrogen peroxide [31]. Therefore, we propose that *PsnSAUR6* likely contributes to drought tolerance by strengthening both the osmotic adjustment and antioxidant systems, thereby mitigating oxidative damage and maintaining cell membrane integrity.

Stomatal aperture regulation is a crucial mechanism for plants to conserve water and enhance drought adaptation [3]. In this study, we found that overexpression of *PsnSAUR6* significantly reduced the stomatal aperture of transgenic tobacco leaves. This effect was more pronounced following drought treatment, resulting in a markedly lower rate of water loss in transgenic lines compared to the wild type (WT). Stomatal closure is a complex process governed by multiple signals, including hormonal signals like ABA, environmental cues such as atmospheric humidity and CO_2_, and the dynamics of intracellular messengers like ROS, NO, and Ca^2+^ within guard cells [3,47]. However, this regulation is primarily mediated by ABA under water-deficient conditions. Building on this understanding, our further experiments confirmed an ABA-related function for *PsnSAUR6*. Specifically, transgenic tobacco seeds exhibited significantly lower germination rates than the wild type under various concentrations of ABA, indicating enhanced ABA sensitivity. Consistent with this, the expression of *PsnSAUR6* in poplar leaves was induced by exogenous ABA treatment, displaying a bimodal pattern with peaks at 0.5 h and 6 h post-treatment. We propose that the first expression peak reflects the involvement of *PsnSAUR6* in the initial, rapid phase of the ABA response, contributing to early stomatal closure. This is corroborated by studies in other species, for instance, *Arabidopsis* mutants lacking *AtSAUR32* show reduced ABA sensitivity and larger stomatal apertures [32], while overexpression of *PtSAUR8* in transgenic *Arabidopsis* enhances drought resistance via ABA-mediated stomatal regulation [31]. The second expression peak at 6 h may be attributed to the involvement of *PsnSAUR6* in a later phase of the ABA response, likely related to stress adaptation processes such as managing oxidative stress. This interpretation is consistent with our earlier physiological measurements and is strongly supported by studies in maize (*Zea mays*). In maize, a single ABA treatment has been shown to induce the bimodal expression of *Zmrbohs* and a corresponding bimodal pattern of NADPH oxidase activity, ultimately leading to biphasic ROS accumulation [48]. Therefore, we propose that *PsnSAUR6* participates in two distinct functional stages of the drought response: an initial phase of stomatal regulation followed by a later phase of oxidative stress management.

Transcriptome profiling further characterized the molecular network downstream of *PsnSAUR6*. A total of 823 DEGs were identified in the overexpression lines. In alignment with the gene functions and physiological traits of transgenic tobacco, these DEGs were significantly enriched in processes including water deficit response, ABA response, oxidative stress management, and transmembrane transport. Notably, we observed the significant downregulation of two genes encoding protein phosphatase 2Cs (*PP2C28* and *PP2C37*), coupled with the significant upregulation of stress-responsive transcription factors—such as *MYB2* (v-myb avian myeloblastosis viral oncogene homolog 2), *ERF020* (Ethylene Response Factor 20), *NAC83* (NAC domain-containing protein 83) and *HAK5* (High-affinity potassium transporter 5).

The Protein Phosphatase 2C (PP2C) family, a highly conserved group of serine/threonine phosphatases, plays a crucial role in stress signaling. In the model plant *Arabidopsis thaliana*, the PP2C family comprises 80 members across 13 subfamilies (A-L). Among these, the clade A subfamily is particularly notable, consisting of members such as ABI1, ABI2, HAB1, HAB2, AHG1, AHG3, HAI1, HAI2, and HAI3 [49]. Except for the HAI proteins, which participate in stress responses through ABA-independent pathways, the remaining members of this clade are core negative regulators of the ABA signaling pathway and negatively regulate plant responses to drought stress. To discern the specific functions of the downregulated genes *PP2C28* and *PP2C37*, we analyzed their *cis*-regulatory elements and phylogenetic relationships with *Arabidopsis* homologs (Figure S2). The *PP2C28* promoter contains numerous MYB-responsive elements but lacks ABA-related motifs, and the protein PP2C28 clusters with the uncharacterized *Arabidopsis* protein AT2G34740. In contrast, the *PP2C37* promoter is enriched in ABA-responsive elements (ABREs), and the protein is a close homolog of AHG3 (AT3G11410), a key repressor of ABA signaling. Previous studies have demonstrated that *Arabidopsis ahg3* mutants display hypersensitivity to ABA during germination, consistent with the phenotype observed in our study [50]. Consequently, we propose that *PP2C37* functions as a negative regulator of the ABA pathway in *PsnSAUR6*-overexpressing tobacco. Its suppression by *PsnSAUR6* appears to relieve the inhibition of ABA signaling, leading to the activation of downstream stress-responsive genes and enhanced drought tolerance.

Transcription factors (TFs) are crucial mediators of plant drought stress responses. The RNA-seq analysis revealed that the expression of *NtaNAC83*, *NtaERF020*, and *NtaMYB2* was significantly elevated in *PsnSAUR6*-overexpressing tobacco. To elucidate their potential regulatory mechanisms, we performed a *cis*-acting element analysis of their promoter regions (Figure 10B). Notably, all three promoters contained ABA-responsive elements (ABREs), suggesting their involvement in ABA-mediated signaling pathways. Furthermore, the promoter of *NtaNAC83* harbored a MYB binding site (MBSI), which is implicated in the regulation of flavonoid biosynthesis. This finding suggests a potential crosstalk, where *NtaNAC83* may cooperate with MYB factors to modulate ABA-regulated antioxidant responses.

The phylogenetic relationship of these upregulated transcription factors with their *Arabidopsis* homologs underscores their potential functional roles. Specifically, NtaMYB2 exhibited the highest homology with AtMYB71 (AT3G24310) (Figure S3A). Previous research has demonstrated that *ATMYB71* is involved in ABA signaling and its overexpression leads to ABA hypersensitivity in seeds [51]. Similarly, the well-characterized *AtMYB2* acts as a transcriptional activator in the ABA signaling pathway, enhancing drought resistance by inducing the expression of stress-responsive genes like *RD22* [52]. Based on this homology, we hypothesize that *NtaMYB2* likely functions within the ABA signaling pathway to regulate downstream drought-responsive genes, thereby contributing to drought tolerance. For NtaERF020, its closest homolog in *Arabidopsis thaliana* is AT1G71520, a member of the *DREB* subfamily of *ERF* transcription factors (Figure S3B). The function of this subfamily in drought tolerance is well-established. For instance, another member, *AtERF019* (the second closest Arabidopsis homolog to *NtaERF020*), enhances drought resistance by promoting stomatal closure and reducing cell wall permeability [53]. Conversely, suppressing the wheat *ERF* transcription factor *TaRAP2-13L* impairs drought resistance by disrupting ABA signaling and ROS homeostasis [54]. Collectively, this evidence suggests that *NtaERF020* is likely involved in the ABA signaling pathway to mitigate oxidative damage under drought stress. *NAC* transcription factors are pivotal regulators of plant drought responses, acting via both ABA-dependent and independent pathways. For instance, *GhirNAC2* in cotton enhances ABA accumulation and promotes stomatal closure [55], while overexpression of *GmNAC065* in soybean has been shown to alleviate oxidative stress and delay senescence [56]. Crucially, our analysis revealed that *NtaNAC83* is highly homologous to ANAC083 (Figure S3C), the *Arabidopsis* ortholog of the aforementioned stress-alleviating *GmNAC065*. This homologous link to a known oxidative stress regulator, combined with the presence of MBSI and ABRE binding sites in the *NtaNAC83* promoter, forms a strong basis for our hypothesis. Therefore, we propose that *NtaNAC83* functions as an ABA-responsive transcription factor that, in concert with MYB proteins, activates the flavonoid pathway to enhance the plant’s capacity for oxidative stress tolerance during drought.

Maintaining potassium (K^+^) homeostasis is critical for plants to cope with drought stress, primarily through its roles in stomatal regulation and osmotic adjustment. For example, the application of potassium to sunflower (*Helianthus annuus* L.) leaves improves gas exchange and antioxidant defenses, thereby stabilizing the plant’s water status under stress [57]. Plants primarily absorb K^+^ from the soil via active transport in root cells, followed by its distribution to various tissues through channel proteins and transporters. Among these, the HAK/KUP/KT family is the most extensively characterized and is ubiquitously expressed across various plant species and tissues [58]. Specifically under low-potassium stress, *HAK* transporters are highly expressed in roots, functioning as the primary system for K^+^ acquisition [59,60]. This function establishes a direct link between the *HAK* family and abiotic stress tolerance, where they are vital for maintaining osmotic pressure and ionic equilibrium during water deficit. Consistent with this role, overexpression of *OsAKT1* in rice increased K^+^ content in leaves and roots, enhancing tolerance to osmotic and drought stress [61]. Similarly, in cotton (*Gossypium hirsutum* L.), SOS1, AKT1, and HAK5 were found to increase the root cellular K^+^/Na^+^ ratio and reduce the over-accumulation of reactive oxygen species (ROS) [62]. In this study, we observed a significant upregulation of *HAK5* expression in the *PsnSAUR6*-overexpressing tobacco lines. The closest ortholog of NtaHAK5 in *Arabidopsis thaliana* is AtHAK5 (AT4G13420), a well-documented transporter responsible for high-affinity K^+^ uptake in roots under potassium-limiting conditions (Figure S3D) [63]. Based on these findings, we hypothesize that *PsnSAUR6* promotes drought tolerance through the transcriptional activation of *HAK5*. This leads to enhanced K^+^ accumulation, which is critical for maintaining cellular turgor and osmotic potential, thereby ensuring plant survival under drought stress.

## 4. Materials and Methods

### 4.1. Plant Materials and Growth Conditions

Poplar (*Populus simonii × P. nigra*) seedlings were provided by Northeast Forestry University. Tobacco plants were *Nicotiana tabacum* L. c.v. Petit HavanaSR-1. Poplar seedlings were cultured in 1/2 MS medium (Coolaber, Beijing, China) and tobacco seedlings were cultured in MS medium (Coolaber, Beijing, China). The tobacco differentiation medium consisted of MS + 1 mg/L 6-BA (Biotopped, Beijing, China) + 0.1 mg/L NAA (Biotopped, Beijing, China); the rooting medium comprised MS + 0.04 mg/L NAA. Culture conditions for poplar and tobacco: Day/night temperature 26 °C/22 °C, 16 h/d illumination at 175 μmol/(m^2^·s), relative humidity approximately 75%.

### 4.2. Cloning and Bioinformatics Analysis of the PsnSAUR6

Total RNA was extracted from leaves of poplar (*Populus simonii × P. nigra*) using the Quick RNA Isolation Kit (Huayueyang, Beijing, China). A total of 1.5 μg of RNA was transcribed into cDNA using the PrimeScript™ RT Reverse Transcription Kit with the gDNA removal function (Takara, Beijing, China) in a final volume of 10 μL. PCR amplification was carried out with cDNA as template, employing KOD-plus-neo high-fidelity enzyme (Toyobo, Osaka, Japan) and primers (Table S1), followed by purification of the product using the Tiangel kit (Tiangen, Beijing, China). The purified PCR product was sent to Genewiz (an Azenta Life Sciences company, Suzhou, China) for sequencing. The obtained sequence was verified as the complete coding sequence of *PsnSAUR6*.

Protein physicochemical properties were analyzed using ProtParam (https://web.expasy.org/protparam/, accessed on 10 July 2025). Secondary structure prediction was performed with GOR4 (https://npsa-prabi.ibcp.fr/cgi-bin/npsa_automat.pl?page=npsa_gor4.html, accessed on 10 July 2025). To identify relevant *cis*-regulatory elements, the 2000 bp promoter region upstream of *PsnSAUR6* was analyzed with the PlantCARE database (https://bioinformatics.psb.ugent.be/webtools/plantcare/html/, accessed on 12 July 2025). Putative elements related to hormone signaling and stress responses were then selected from the results and visualized using TBtools v2.420 for further analysis. Based on BLAST results from NCBI (E-value < 1 × 10^−10^), homologous amino acid sequences across plant species were retrieved for PsnSAUR6 (https://blast.ncbi.nlm.nih.gov/Blast.cgi, accessed on 26 July 2025). These sequences were aligned using MUSCLE, from which a phylogenetic tree was inferred in MEGA 11 under the Neighbor-Joining (NJ) model with 1000 bootstrap replicates for node support estimation.

### 4.3. Expression Analysis of PsnSAUR6 Under PEG6000-Induced Osmotic Stress

Cuttings from a single clone of poplar, harvested from a greenhouse, were cultivated hydroponically. After the new roots, stems and leaves (approximately 40 days) grew on the poplar branches, the plants were divided into two groups. One group was maintained under normal watering as the control, while the other was treated with a 20% (*w*/*v*) PEG6000 solution (Guangfu, Tianjin, China) to induce osmotic stress (Ψ ≈ −0.7 MPa at 25 °C, calculated according to Michel [64]). Polyethylene glycol (PEG6000) is a synthetic polymer whose high molecular weight prevents it from penetrating the plant cell wall. Thus, the cytorrhysis induced by PEG6000 closely mimics the physical stress of soil drought. Furthermore, its minimal absorption and low cytotoxicity ensure the stress is primarily osmotic [65]. Root (new root tips), stem (the third internode from the apex), and leaf (the second mature leaf from the apex) tissues were sampled from both groups at 0 h, 6 h, 12 h, 24 h, 48 h and 72 h post-treatment. The PEG6000 concentration and sampling time points were identical to those used in our previous transcriptome analysis of poplar (unpublished data). Total RNA was extracted from these tissues, followed by reverse transcription and quantitative real-time PCR (qRT-PCR) analysis to examine the expression patterns of the *PsnSAUR6* gene in different tissues under different conditions.

The qRT-PCR reactions were carried out in a 10 µL volume containing 1 µL cDNA, 0.4 µL each of forward and reverse primers, and 5 µL SYBR Premix EX Taq™ II (Tli RNase H Plus) (Bao Bio, Dalian, China). All reactions were performed in triplicate. Amplification and detection were conducted on a BIO-RAD system using the following thermal profile: initial denaturation at 95 °C for 2 min, followed by 33 cycles of denaturation at 95 °C for 30 s, and annealing/extension at 55 °C for 30 s. For data normalization, poplar samples used the *ACTIN* gene as an internal reference, while tobacco samples were normalized against both *UBQ* and *EFI* genes. Relative expression levels were calculated using the 2^–ΔΔCt^ method, with the control group set to 1.0. The sequences of gene-specific primers used for qRT-PCR are listed in Table S1.

### 4.4. Transformation and Cultivation of Transgenic Tobacco

Tobacco transformation and cultivation were performed following protocols [66]. The verified coding sequence of *PsnSAUR6* (with terminator) was ligated into the pROKII vector (provided by Northeast Forestry University), which contains the CaMV 35S promoter. The recombinant plasmid pROKII-*PsnSAUR6* was first transformed into *Escherichia coli DH5α* competent cells. Following antibiotic selection and PCR-based screening, positive clones were validated by sequencing at Genewiz (an Azenta Life Sciences company, Suzhou, China). The verified plasmid was extracted using the E.Z.N.A.^®^ Plasmid Mini Kit I (Omega Bio-tek, Norcross, GA, USA) and subsequently introduced into *Agrobacterium tumefaciens EHA105* via electroporation. Tobacco (*Nicotiana tabacum* cv. SR-1) was then transformed using the leaf-disk method. After co-cultivation for two days, the infected leaves were transferred to fresh differentiation medium supplemented with 6-BA and NAA. Tobacco leaf genomic DNA was extracted using the CTAB method for PCR-based preliminary screening to verify the successful integration of the recombinant vector. Total RNA was extracted from leaves of T_2_ generation plants and analyzed by quantitative real-time PCR to determine *PsnSAUR6* expression levels. The lines with a higher expression of *PsnSAUR6* were selected, and harvested seeds of those lines (T_3_ seeds) were used for further experiments.

### 4.5. Phenotypic Analysis of Transgenic Tobacco

#### 4.5.1. Survival Rate and Biomass Assessment Under Drought Stress

Seeds of non-transgenic wild-type (WT) tobacco and T_3_ generation *PsnSAUR6* transgenic tobacco lines were germinated on MS medium. One week after germination, when cotyledons were fully expanded, 24 uniformly grown seedlings per line were transplanted into pots (15.7 × 13.5 × 11 cm, 2.1 L) filled with a nutrient soil mixture (vermiculite: potting mix = 2:1) and watered with 750 mL every three days.

For drought treatment, four-week-old plants were subjected to a 12-day drought period by withholding water, followed by a 5-day recovery period with normal irrigation. A plant was considered to have survived if new green leaves emerged after rewatering. Survival rate was calculated as: (Number of surviving plants/Total number of treated plants) × 100%. Each line was assessed with three biological replicates.

Phenotypes were recorded pre-drought, post-drought, and post-recovery. Three biological replicates were performed. For each tobacco strain, three plants (biological replicates) were randomly harvested at two time points: before the initiation of drought treatment and after 12 days of drought stress. The fresh weight and dry weight of the aerial parts were subsequently determined.

#### 4.5.2. Assessment of Root Growth Under Osmotic Stress

Seeds of WT and T_3_ generation *PsnSAUR6* transgenic tobacco were germinated on MS medium. One week after germination, once the cotyledons had fully expanded, seedlings of uniform size from each line were selected and divided into two groups. One group was transferred to fresh MS medium to serve as a control, while the other was subjected to osmotic stress. Although PEG6000 was initially tested, it was found to interfere with the solidification of the MS medium. Furthermore, preliminary trials using PEG-infused agar plates [67] yielded inconsistent results across replicates and failed to produce distinct phenotypic changes. Consequently, following the method of Zhang et al. [68], 200 mM mannitol (Biotopped, Beijing, China) was employed to simulate osmotic stress (Ψ ≈ −0.5 MPa at 25 °C, calculated according to Michel [69]) for the treatment group. Both groups were cultivated vertically in a growth chamber under controlled conditions (25 ± 2 °C, 75% relative humidity, and a 16 h light/8 h dark photoperiod). After a further 10 days of vertical incubation, the primary root lengths of seedlings in each group were measured. Three biological replicates were performed for each line.

#### 4.5.3. Quantification of Antioxidant Enzyme Activity and Osmoprotectants

Wild-type tobacco and T_3_ generation transgenic tobacco seeds were sown on MS medium. One week later, tobacco seedlings with similar growth conditions from each strain were transplanted to trays (54 × 28 cm, 110 mL per well, 32 wells total) for substrate (potting mix: perlite: peat (Peyres) = 2: 1: 1). Each tray was watered with 1.3 L every four days. At the 3-week growth stage, different strains with similar growth conditions were selected for stress treatment. The drought stress group received no watering for 7 days, while the control group continued normal watering. The fifth fully expanded true leaf from the top was collected from different plants for physiological parameter analysis.

Peroxidase (POD) activity, malondialdehyde (MDA) content, and proline content were determined using specific assay kits (Solarbio, Beijing, China) with three biological replicates per line. POD activity was measured using the guaiacol oxidation method. One unit (U) of POD activity was defined as the amount of enzyme causing an increase of 0.005 in absorbance at 470 nm per minute per gram of fresh tissue [70,71]. The MDA content was estimated based on thiobarbituric acid reactive substances (TBARS) using the TBA method. Briefly, under acidic and high-temperature conditions, MDA reacts with TBA to form a colored complex. To minimize interference from non-specific substances, absorbance was measured at both 532 nm and 600 nm. The concentration of TBARS was calculated based on the difference in absorbance (A_532_–A_600_) and used to estimate MDA content, expressed as nmol/g fresh weight (FW) [72,73]. Proline content was determined using the acidic ninhydrin method. Proline was extracted with sulfosalicylic acid and reacted with acidic ninhydrin under heating to produce a red complex. The absorbance was measured at 520 nm, and the content was calculated against a standard curve and expressed as μg/g FW [74,75]

#### 4.5.4. Measurement of Detached Leaf Water Loss Rate

At four weeks post-planting, tobacco plants (prepared according to Section 4.5.3) were sampled. The uppermost three to four fully expanded leaves from each plant, collected from the same nodal position and with similar growth status, were collected. The leaf water-loss rate was determined according to the protocols described by Liu et al. [76] and Negi et al. [77]. Water loss percentage was calculated as [(WF − WT)/(WF − WD)] × 100%, where WF represents the initial fresh weight, WT is the fresh weight at each measurement time point, and WD is the final dry weight. Fresh weight was recorded every 2 h after leaf detachment until the 12th hour, and leaves were then oven-dried at 80 °C for 2–3 days to obtain the dry weight. Measurements were conducted under controlled conditions of 25 °C and 50 ± 5% relative humidity. The experiment was performed with three biological replicates.

#### 4.5.5. Measurement of Stomatal Aperture and Opening Rate

Stomatal analysis was performed on fully expanded leaves from four-week-old, well-watered plants grown as described in Section 4.5.3. Leaves were selected from identical nodal positions from both WT and transgenic *PsnSAUR6* lines. The nail polish replica method [78] was used to observe the stomatal aperture (diameter) and the proportion of open stomata (opening rate) were quantified in a microscope system equipped with a high-speed camera (MDX4-T, Minami, Guangdong, China). For each line, the aperture was measured for at least 50 stomata to calculate the mean value, and the opening rate was determined from randomly selected fields of view of equal area across different leaves. The experiment was conducted in triplicate.

### 4.6. The Response of PsnSAUR6 to ABA

#### 4.6.1. Effect of ABA on *PsnSAUR6* Expression

One-month-old tissue-cultured poplar seedlings were sprayed with a solution containing 100 μM ABA (Coolaber, Beijing, China) and 0.5% (*v*/*v*) Triton X-100 until a fine mist uniformly covered the leaf surface without runoff. The 2nd to 4th uppermost leaves were sampled at 0 h, 0.5 h, 1 h, 2 h, 4 h, 6 h, and 12 h post-treatment. Total RNA was extracted, reverse-transcribed into cDNA, and subjected to quantitative real-time PCR analysis as described in Section 2.4 to examine the temporal expression profile of the *PsnSAUR6* gene in response to ABA.

#### 4.6.2. ABA-Mediated Inhibition of Seed Germination

The transgenic tobacco and the wild-type tobacco seeds were respectively sown on MS solid culture medium containing ABA (0 μM, 1 μM, 3 μM) for 8 days. A seed was scored as germinated upon full cotyledon expansion. The germination rate was calculated as: (Germinated seeds/Total seeds) × 100%. Three experimental replicates were performed.

### 4.7. Transcriptome Analysis of Transgenic Tobacco Under PEG6000-Induced Osmotic Stress

For transcriptome analysis, two-week-old seedlings of WT and transgenic lines were harvested after 48 h treatment with PEG6000 (−0.7 MPa) [79], a stage when osmotic stress symptoms were evident but not severe. Three biological replicates per line were sequenced by Genewiz (an Azenta Life Sciences company, Suzhou, China). Differentially expressed genes (DEGs) were identified using R, followed by Gene Ontology (GO) and Kyoto Encyclopedia of Genes and Genomes (KEGG) pathway enrichment analyses. Visualization was conducted with the following R packages: ggplot2 for volcano plots, enrichplot for bar plots of GO/KEGG terms, and GOplot for GO chord diagrams. Selected DEGs associated with drought resistance, as identified by transcriptomics, were further validated by quantitative real-time PCR (Section 2.4) to compare their expression patterns between transgenic and wild-type plants.

### 4.8. Statistical Analysis and Data Visualization

To ensure experimental reliability, all experiments were replicated for validation. Data analysis was performed using IBM SPSS Statistics 25 software with Duncan’s Multiple Range (DMR) test. Data in figures are presented as the mean ± standard deviation (± SD) from three independent experiments. Asterisks indicate statistically significant differences compared to non-transgenic (WT): (* *p* <0.05; ** *p* <0.01; *** *p* < 0.001). All figures were generated using GraphPad Prism 8.0 software.

## 5. Conclusions

In this study, the small auxin up RNA (*SAUR*) gene *PsnSAUR6* was cloned from *Populus simonii × P. nigra*, and its physicochemical properties were characterized. Transcriptional analysis revealed that *PsnSAUR6* exhibits organ-specific expression patterns; its expression is induced by drought stress in stems and leaves but is suppressed in roots. Overexpression of *PsnSAUR6* in tobacco significantly enhanced its drought tolerance.

Based on subsequent physiological and transcriptomic analyses, we propose a multi-faceted model for how *PsnSAUR6* confers drought resistance: (1) *PsnSAUR6* promotes biomass accumulation and primary root elongation under drought stress. This likely expands the plant’s water acquisition capacity, enabling it to better withstand water-deficit conditions. (2) *PsnSAUR6* initiates a signaling cascade by repressing the expression of the phosphatase PP2C37, which in turn activates the ABA signaling pathway. This leads to the induced expression of transcription factors (*NAC83*, *ERF020*, and *MYB2*) and other downstream stress-responsive genes. Ultimately, this ABA-dependent mechanism promotes stomatal closure, thereby reducing water loss and enhancing the plant’s drought tolerance. (3) Under drought stress, *PsnSAUR6* elevates both proline content and POD activity, which mitigates oxidative damage and thus enhances the plant’s drought tolerance. (4) *PsnSAUR6* upregulates the expression of the high-affinity potassium transporter *HAK5*, which may contribute to maintaining cellular turgor and osmotic potential under drought stress by enhancing K^+^ accumulation.

Against the backdrop of intensifying global climate change and the severe drought stress facing forest ecosystems, this study provides the first evidence that the auxin-responsive gene *PsnSAUR6* acts as a positive regulator of drought tolerance in *Populus simonii × P. nigra*. We further demonstrate its responsiveness to abscisic acid (ABA) and propose a mechanism whereby *PsnSAUR6* synergistically enhances drought resistance by integrating ABA pathways. This discovery not only deepens our understanding of hormonal crosstalk in woody plant adaptation to adverse conditions but also offers a novel strategy for stress-resistant tree breeding. By targeting this endogenous auxin signaling hub, it may be possible to overcome the canonical trade-off between stress defense and growth, thereby developing new cultivars that can effectively cope with drought while maintaining robust growth and carbon sequestration potential. These findings provide a crucial theoretical foundation and innovative breeding approaches for adapting to climate change, ensuring forest ecological security, and promoting sustainable forestry.

## Figures and Tables

**Table 1:** Comparison of leaf fresh weight (FW) and dry weight (DW) among tobacco genotypes under control conditions.

Genotype	Fresh Weight (g)	Dry Weight (g)	CV of Fresh Weight (%)	CV of Dry Weight (%)
WT	0.0347 ± 0.0069	0.0072 ± 0.0012	19.87%	16.49%
OE-2	0.0292 ± 0.0058	0.0068 ± 0.0013	19.90%	19.29%
OE-3	0.0257 ± 0.0039	0.0058 ± 0.0005	15.07%	9.12%

Data are presented as the mean ± SD (*n* = 3). A coefficient of variation (CV) < 20% was considered indicative of statistically significant variation among replicates.

**Table 2:** RNA-seq Results of Candidate Genes.

Gene Name	log_2_FoldChange	*p*-Value	padj	Description
*MYB2*	3.11	1.34 × 10^−5^	2.48 × 10^−3^	Transcription factor MYB2
*ERF020*	2.82	6.61 × 10^−4^	3.35 × 10^−2^	Ethylene-responsive transcription factor ERF020
*HAK5*	1.70	1.17 × 10^−3^	4.77 × 10^−2^	Potassium transporter 5
*NAC83*	1.27	7.44 × 10^−4^	3.60 × 10^−2^	NAC domain-containing protein 83
*PP2C 28*	−3.14	9.71 × 10^−7^	3.65 × 10^−4^	Probable protein phosphatase 2C 28
*PP2C 37*	−2.04	9.39 × 10^−6^	1.89 × 10^−3^	Protein phosphatase 2C 37

## Data Availability

The original contributions presented in this study are included in the article/Supplementary Materials. Further inquiries can be directed to the corresponding authors.

## Supplementary Materials

The following supporting information can be downloaded at: https://www.mdpi.com/article/10.3390/plants15091398/s1, Figure S1: Phenotypic diagram of stomatal responses to drought stress in transgenic *PsnSAUR6* tobacco and non-transgenic tobacco; Figure S2. Phylogenetic analysis of NtaPP2C28 and NtaPP2C37 with their *Arabidopsis thaliana* homologs; Figure S3. Phylogenetic analysis of selected upregulated genes identified in transcriptomics; Table S1: Primers used in this study; Table S2: Physicochemical Properties of PsnSAUR6 Protein; Table S3: Comparison of stomatal opening rate tobacco genotypes under control conditions.
