# Supplementary material for: The Small Auxin Upregulated RNA PsnSAUR6 from Populus simonii × P. nigra Enhances Drought Tolerance in Transgenic Tobacco
Source: Plants (Basel). 2026 May 2;15(9):1398. doi: 10.3390/plants15091398 (PMC13164708; doi:10.3390/plants15091398)
Supplement: Supplementary file 1 [file plants-15-01398-s001.zip › plants-4273893-supplementary.pdf]

The amino acid sequence encoded by the *PsnSAUR6* gene is as follows：
MSGIVRKLWCCGAKGFPSADDSAEDQLALPPPEGHVRVCVGKDNVQCRFEMEAHFLNHP
LFEDLLRLSEQEHGYAYDGALRIACEIHLFQYLLHLLKTGNPTAHYMQLPDLISTFHSSAAH
HKYPPPPPPPPLLNIPPCQYSH*

Table S1 Primers used in this study.

**Primer names**	**Sequence (5′-3′)**	**Description**
PsnSAUR6-F1	TGCTCTAGAATGAGTGGAATCGTTA	Gene cloning
PsnSAUR6-R1	TCCCCCCGGGAAGCAGACCTACAAT	Gene cloning
PsnSAUR6-F2	GCAGGTTTGAGATGGAAGCC	qRT-PCR
PsnSAUR6-R2	TCGCAGGCAATCCTTAAAGC	qRT-PCR
35S-F	GACGCACAATCCCACTATCC	Gene cloning
ACTIN-F	CAGTGTCTGGATTGGAGGGT	qRT-PCR
ACTIN-R	CCGCACTGTTCGGAACTTAG	qRT-PCR
EFI-F	GAATCTGGTCTCAAGTCCGTTA	qRT-PCR
EFI-R	TTCCTCCTCTGTCTCTTCTCC	qRT-PCR
UBQ-F	CCAAGATTCAGGACAAGGAAGG	qRT-PCR
UBQ-R	CACCATCAGGAGCAAGTTAGG	qRT-PCR
HAK5-F	GTTCTCTCGGCCGTAAGTGG	qRT-PCR
HAK5-R	TGCAAATGGCTGGAGCAAATG	qRT-PCR
ERF020-F	AAGCTGCTGCTGTTGCACAT	qRT-PCR
ERF020-R	TGCACTGACCTCGGTGACAT	qRT-PCR
NAC83-F	CCTGGGATTTGCCTGGTGAT	qRT-PCR
NAC83-R	TGGTTTCCAACAGCCACTTG	qRT-PCR
PP2C28-F	TGCCCTGGAAACGGGTCTAA	qRT-PCR
PP2C28-R	AACCTCTCTGCCTGCTCCAT	qRT-PCR
PP2C37-F	CCAATTGCGGTGATTCTCGT	qRT-PCR
PP2C37-R	TAACACGACCACCTGCTTCT	qRT-PCR
MYB2-F	AAAGCTGTAGGTTGAGATGGGT	qRT-PCR
MYB2-R	TTCTCCCTGGCAAGCCTCTA	qRT-PCR

Table S2. Physicochemical Properties of PsnSAUR6 Protein

**Formula**	**Molecular weight (Da)**	**Total number of atoms**	**Theoretical pl**	**GRAVY**	**Instability index（Π）**	**Aliphaticindex**	**Extinction coefficients ((mg/mL)$^{-1}$ cm^{-1})**
$C_{725}H_{1094}N_{196}O_{201}S_9$	16060.39	2225	6.08	-0.301	51.75	80.56	0.922

Table S3. Comparison of stomatal opening rate tobacco genotypes under control conditions.

Condition	Genotype	Stomatal opening rate 1 (%)	Stomatal opening rate 2 (%)	Stomatal opening rate 3 (%)	Average pore stomatal opening rate (%)
Control	WT	60	55.35	51.52	55.62
	OE-2	32.35	38	34.15	34.83
	OE-3	34.88	37.14	32.56	34.86
Drought	WT	33.33	37.04	42.42	37.60
	OE-2	26.08	31.25	27.5	28.28
	OE-3	28.57	22.72	27.2	26.16

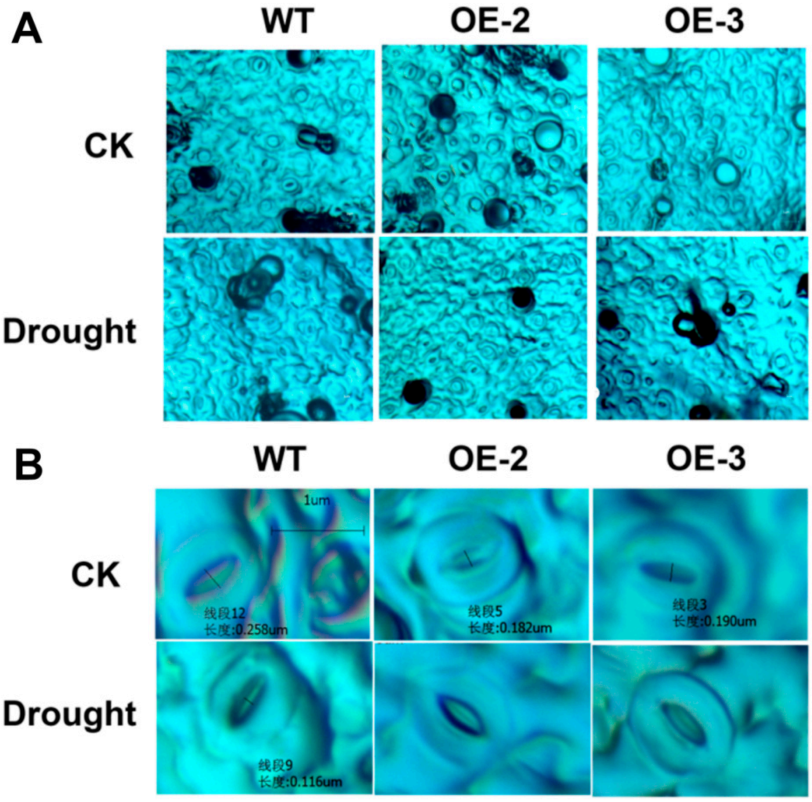

Figure S1. Phenotypic diagram of stomatal responses to drought stress in transgenic *PsnSAUR6* tobacco and non-transgenic tobacco. A: Microscopic observation of open stomata in non-transgenic and transgenic tobacco, with a scale of 1 μm; B: Microscopic observation of stomatal aperture in non-transgenic and transgenic tobacco, with a scale of 1 μm. (WT: Non-transgenic tobacco; OE-2 and OE-3: Different transgenic lines)

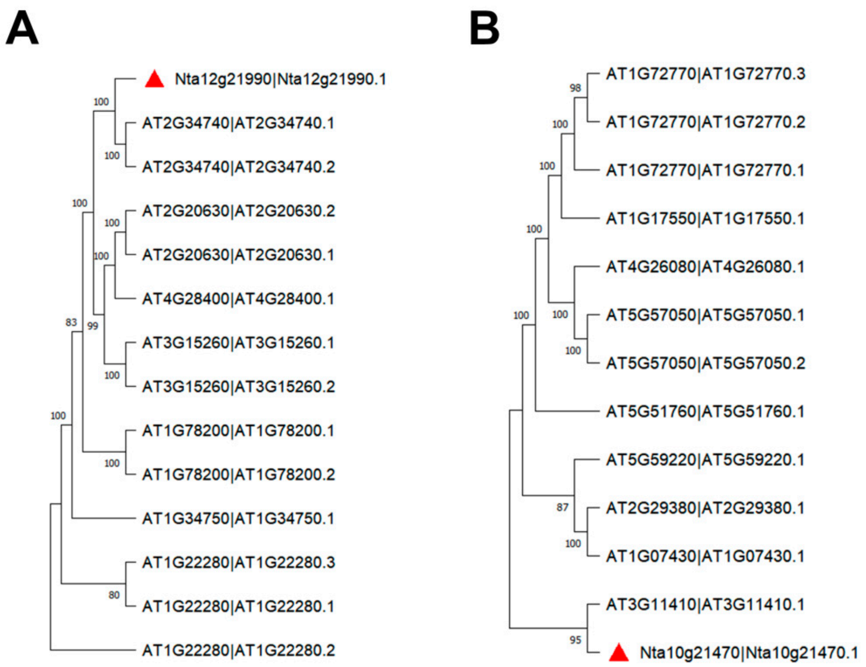

Figure S2. Phylogenetic analysis of NtaPP2C28 and NtaPP2C37 with their *Arabidopsis thaliana* homologs. A: Phylogenetic tree of NtaPP2C28 and its *A. thaliana* homologs. The red triangle marks NtaPP2C28. B: Phylogenetic tree of NtaPP2C37 and its *A. thaliana* homologs. The red triangle marks NtaPP2C37. Methodological note: Homologs were selected from BLAST results with E-value < 1×10^{-10} and identity ≥ 45%. Trees were constructed by the Neighbor-Joining method in MEGA11, with genetic distances computed using the *p*-distance model. Node stability was assessed with 1000 bootstrap replicates; only support values ≥ 50% are shown. Scale bar indicates amino acid *p*-distance per site.

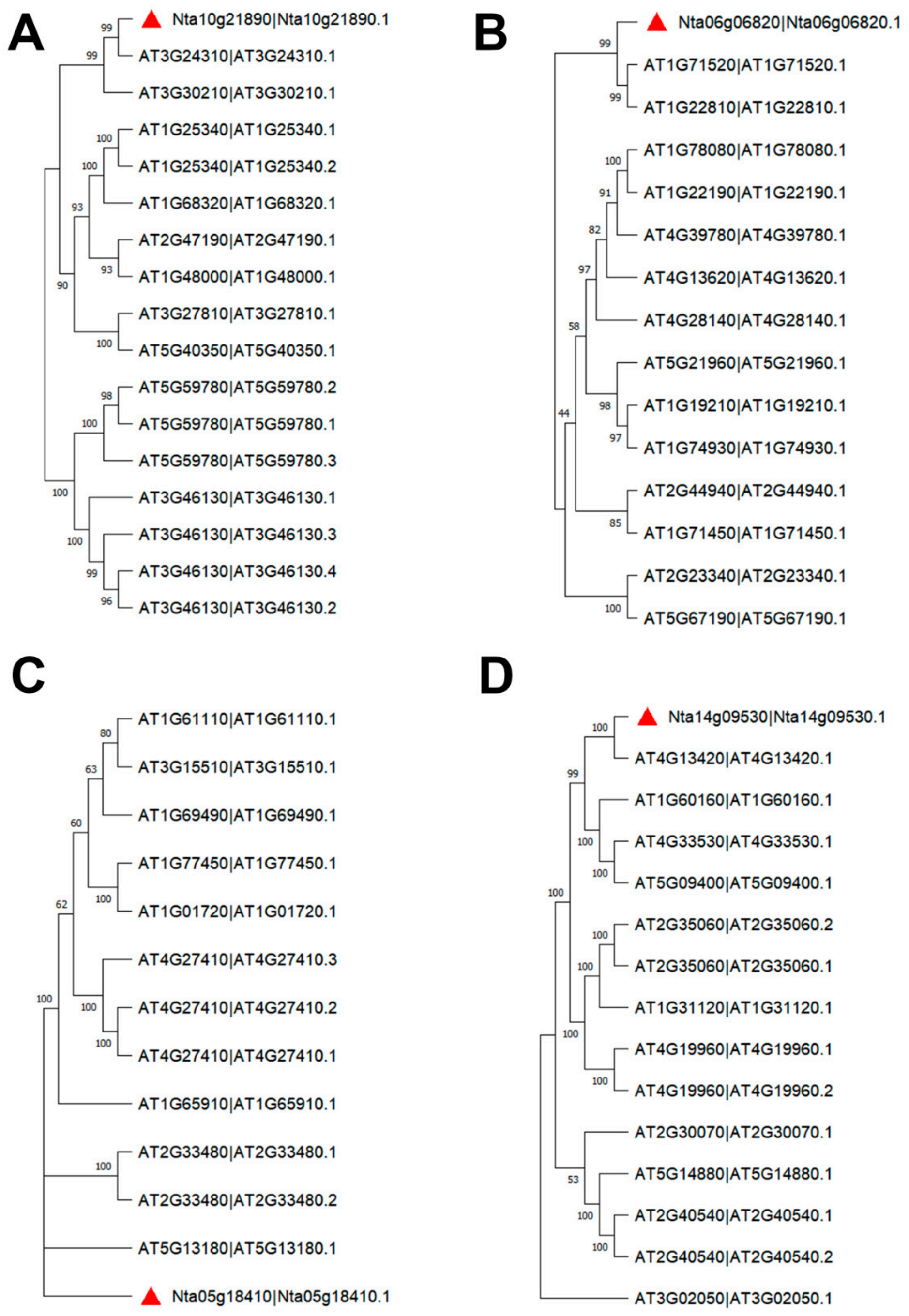

Figure S3. Phylogenetic analysis of selected upregulated genes identified in transcriptomics. A: NtaMYB2 and its *Arabidopsis* homologs (E-value $<1 \times 10^{-45}$, identity ≥ 60%). B: NtaERF020 and its *Arabidopsis* homologs (E-value $<1 \times 10^{-10}$, identity ≥ 45%). C: NtaNAC83 and its *Arabidopsis* homologs (E-value < 1e-10, identity ≥ 45%). D: NtaHAK5 and its *Arabidopsis* homologs (E-value $< 1 \times 10^{-10}$, identity ≥ 40%). In all panels, the red triangle marks the corresponding tobacco gene. Methodology: Trees were constructed by the Neighbor-Joining method in MEGA11, with genetic distances computed using the *p*-distance model. Node support was evaluated with 1000 bootstrap replicates; only values ≥ 50% are shown. Scale bar indicates amino acid *p*-distance per site.
